# Thermal hysteresis measurement of the VO_2_ emissivity and its application in thermal rectification

**DOI:** 10.1038/s41598-018-26687-9

**Published:** 2018-05-31

**Authors:** C. L. Gomez-Heredia, J. A. Ramirez-Rincon, J. Ordonez-Miranda, O. Ares, J. J. Alvarado-Gil, C. Champeaux, F. Dumas-Bouchiat, Y. Ezzahri, K. Joulain

**Affiliations:** 1 Departamento de Física Aplicada, Cinvestav-Unidad Mérida, Carretera Antigua a Progreso km. 6, 97310 Mérida, Yucatán Mexico; 2 Institut Pprime, CNRS, Université de Poitiers, ISAE-ENSMA, F-86962 Futuroscope Chasseneuil, France; 3 Université de Limoges, CNRS, IRCER, UMR 7315, F-87000 Limoges, France

## Abstract

Hysteresis loops in the emissivity of VO_2_ thin films grown on sapphire and silicon substrates by a pulsed laser deposition process are experimentally measured through the thermal-wave resonant cavity technique. Remarkable variations of about 43% are observed in the emissivity of both VO_2_ films, within their insulator-to-metal and metal-to-insulator transitions. It is shown that: i) The principal hysteresis width (maximum slope) in the VO_2_ emissivity of the VO_2_ + silicon sample is around 3 times higher (lower) than the corresponding one of the VO_2_ + sapphire sample. VO_2_ synthesized on silicon thus exhibits a wider principal hysteresis loop with slower MIT than VO_2_ on sapphire, as a result of the significant differences on the VO_2_ film microstructures induced by the silicon or sapphire substrates. ii) The hysteresis width along with the rate of change of the VO_2_ emissivity in a VO_2_ + substrate sample can be tuned with its secondary hysteresis loop. iii) VO_2_ samples can be used to build a radiative thermal diode able to operate with a rectification factor as high as 87%, when the temperature difference of its two terminals is around 17 °C. This record-breaking rectification constitutes the highest one reported in literature, for a relatively small temperature change of diode terminals.

## Introduction

The capability of manipulating heat to control and process information via phonons, photons, and electrons is of critical importance to efficiently manage the energy resources of nature^1^. Inspired by the ability of the electronic diode to rectify electrical currents, different models of thermal diodes have been conceived and theorized over the last decade. The working principle of this thermal device is based on the asymmetry of the heat flux exchanged between its two terminals when their temperature difference is reversed^2^. This thermal asymmetry is usually characterized by the rectification factor $R=|{q}_{F}-{q}_{B}|/\,{\rm{\max }}({q}_{F};{q}_{B})$, where *q*_*F*_ and *q*_*B*_ stand for the magnitudes of the heat fluxes in the forward and the backward configurations, respectively, for a given temperature difference between the thermal diode terminals. The rectification of conductive heat fluxes is the result of the nonlinearity in the phonon^3–8^ and electron^9,10^ heat channels and was experimentally observed with *R* > 20% in carbon and boron nanotubes^11^, semiconductor quantum dots^12^, nanoribbons^5,13^, reduced graphene oxide^14^ and bulk cobalt oxides^15^.

Thermal rectification through photons driving radiative heat fluxes in both the far^16–18^ and near^19–21^ fields has also been studied widely. Far-field radiative thermal diodes, involving terminals of selective emitters^16^ and superconducting materials^17^ operating with rectification factors up to 70% have been proposed for a temperature difference of 200 K between terminals. In the near-field regime, on the other hand, Iizuka and Fan^19^ found a rectification factor *R* = 44%, for a thermal diode with terminals of coated and uncoated SiC at temperatures of 500 K and 300 K, respectively. These values of the rectification factor in the far and near fields are determined by the terminals permittivity and they have been enhanced up to 90% in radiative thermal diodes capitalizing on the strong permittivity contrast of vanadium dioxide (VO_2_)^22–24^. This phase-change material (PCM), with a strongly correlated electron system, is thus very promising for controlling heat currents due to its reversible metal-insulator transition (MIT) with thermal hysteresis in a small temperature range near room temperature. Below 341 K, VO_2_ exhibits an insulating monoclinic phase that becomes a metallic rutile one above ~346 K^25,26^, which induces sizeable changes on its electrical, optical, and thermal properties. These changes on the VO_2_ properties have allowed the development of models for radiative thermal transistors^27–31^, thermal memories^32^, microwave switches^33,34^, etc.

The aspiration of applying VO_2_ in devices with optimal performances has led to a progressive study and tailoring of the VO_2_ physical properties through its MIT. Several works on the electrical resistivity^35–39^ and electrical conductivity^40^ of single crystals and thin films of VO_2_, reported variations of four and five orders of magnitude, respectively. Yang *et al*.^39^ showed that the width of the hysteresis loop of the VO_2_ electrical resistivity, sharpness of the transition, and transition temperature, depend strongly on the epitaxial growth of VO_2_ films on sapphire. This indicates that the substrate, as well as the growth process used, represent an effective way to tailor the overall properties of VO_2_ films. On the other hand, thermal conductivity variations of VO_2_ films as large as 60% were measured by ultrafast pump-probe techniques^41,42^. The optical transmittance, reflectance, and absorption of VO_2_ films were also measured within its MIT^43^, such that very high absorptions (~ 99.75%) were observed for film thicknesses even smaller than the incident wavelength λ = 11.6 µm. Furthermore, the VO_2_ emissivity was measured indirectly, inside^44^ and outside^45^ the MIT, by means of infrared transmittance and reflectance measurements. Leahu *et al*.^46^ quantified an emissivity hysteresis of about 8 K for a VO_2_ thin film deposited on a silicon wafer and showed that the emissivity variations determined through reflectance measurements in the spectral range (2.5–5.0) µm and on the side of the Si wafer (${\rm{\Delta }}{\epsilon }=\,0.47\,$) are twice larger than those on the VO_2_ side. In addition, these latter authors also observed an anomalous absorption phenomenon through the presence of an emissivity maximum during the heating and cooling processes of VO_2_. The effects of the film thickness, substrate material, and multilayer structures, on the emissivity of the VO_2_ films have also been studied indirectly^47–50^. Given that these indirect measurements of the VO_2_ emissivity require the a priori knowledge of other optical parameters, it is desirable to measure it by means of more accurate experimental methodologies, that allow a deeper comprehension of the involved phenomena occurring during the MIT.

In this paper, we directly measure the principal and secondary hysteresis loops of the VO_2_ emissivity during the heating and cooling processes around its MIT. This is done through the thermal-wave resonant cavity technique (TWRC), for two types of VO_2_ thin films deposited on r-sapphire and silicon (100) substrates by means of the pulsed laser deposition (PLD) process. We show that the substrate has a significant impact on the growth of the VO_2_ thin films and therefore on their overall emissivity, such that the sample with the silicon substrate yields slower MITs and wider hysteresis loops than the one deposited on sapphire. Furthermore, we find that the width along with the rate of change of the VO_2_ emissivity can be tuned through secondary hysteresis loops. Finally, the experimental values of the VO_2_ emissivity are applied to theoretically assess the rectification factor of a radiative thermal diode based on VO_2_.

## Methods and Materials

### Sample fabrication

**Figure 1:**
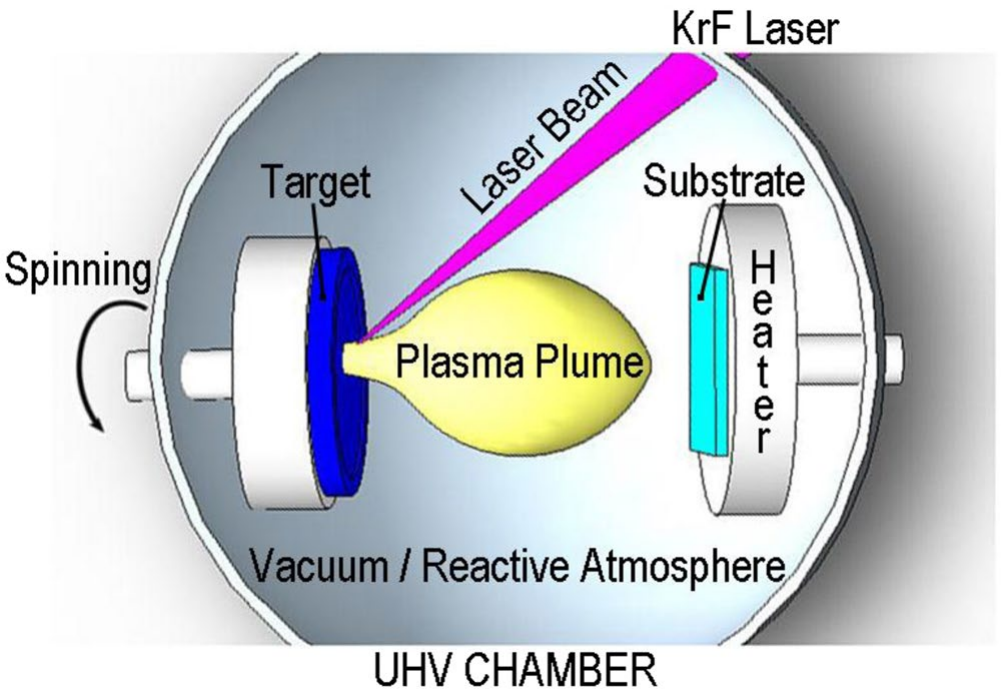
Scheme of the working principle of the pulsed laser deposition (PLD) process.

Vanadium dioxide thin films have been deposited over substrates of r-sapphire and silicon (100) wafers through the PLD process. This well-adapted method for the deposition of complex multi-element materials and oxides consists in using a pulsed high-power laser beam to evaporate a small amount of matter from a solid target within a stainless-steel ultra-high vacuum chamber, as shown in Fig. 1. After some hundreds or thousands of laser pulses, the evaporated matter condensed on a substrate forms a thin film. A KrF pulsed excimer laser with wavelength λ = 248 nm, pulse width of 25 ns, and repetition rate of 25 Hz has been employed to grow VO_2_ films on r-sapphire and silicon (100) substrates set at the temperature of 600 °C. The oxygen pressure used for the deposition of all samples has been of 2.2 Pa. The main characteristics of the substrates and VO_2_ thin films are summarized in Table 1.

**Table 1:** Main characteristics about the substrates and VO_2_ film properties.

	Sample 1	Sample 2
Substrate	r-sapphire	silicon (100)
Dopant and doping concentration	—	P-type: Boron 7 × 10^14^ to 1 × 10^16^ at/cm^3^
Substrate thickness L1 (µm)	510	525
VO_2_ thin film thickness L2 (nm)^a^	120	300
Emissivity (at room temperature)^b^, ${{\boldsymbol{\epsilon }}}_{{\bf{1}}{\boldsymbol{amb}}}$	0.59	0.52

^a^Measured by profilometry (KLA-Tencor AlphaStep D-120), ^b^Determined by a PerkinElmer Frontier FTIR spectrometer in the wavelength range of 2–22 μm, as detailed in the supplementary material.

### Sample characterization

**Figure 2:**
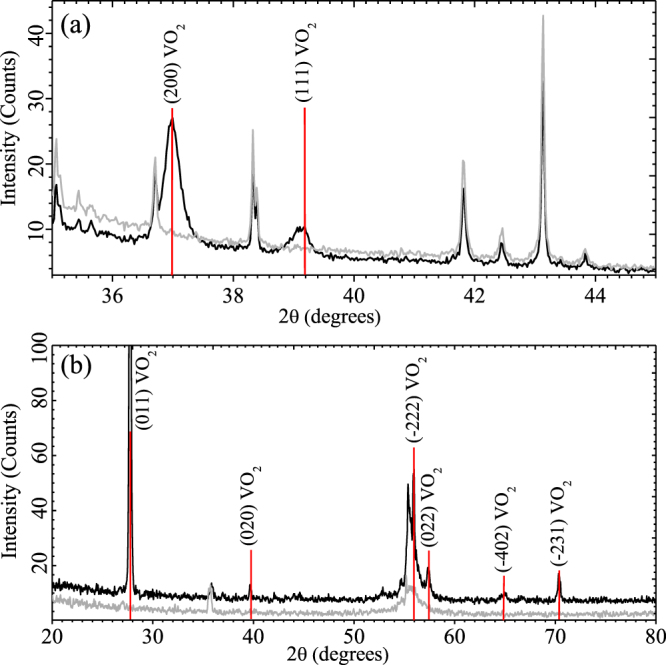
(Black line) Room temperature XRD patterns of VO_2_ films grown on (**a**) r-sapphire (sample 1) and (**b**) silicon 100 (sample 2). Gray lines stand for XRD patterns of the substrates.

The crystalline structure of our VO_2_ samples has been analyzed by X-Ray Diffraction (XRD), whose patterns are shown in Fig. 2. The XRD pattern of sample 1 has been determined through a diffractometer D-8 Advance working with a Bragg-Brentano geometry and CuK_α1_ radiation (Fig. 2a), while that of sample 2 (Fig. 2b) has been found by using a diffractometer Siemens D-5000 (θ, 2θ) operating with an small angle and CuK_αa_ radiation, due to the relatively high intensity of its peaks. According to the International Centre for Diffraction Data (Card 04–003–2035), the data of both samples are found to be consistent with the monoclinic phase of VO_2_ (red lines in Fig. 2), as expected. The VO_2_ film deposited on r-sapphire (Fig. 2a) presents an orientation at the (200) and (111) diffraction peaks, while the one deposited on silicon (Fig. 2b) is not oriented and shows a principal peak in the plane (011).

**Figure 3:**
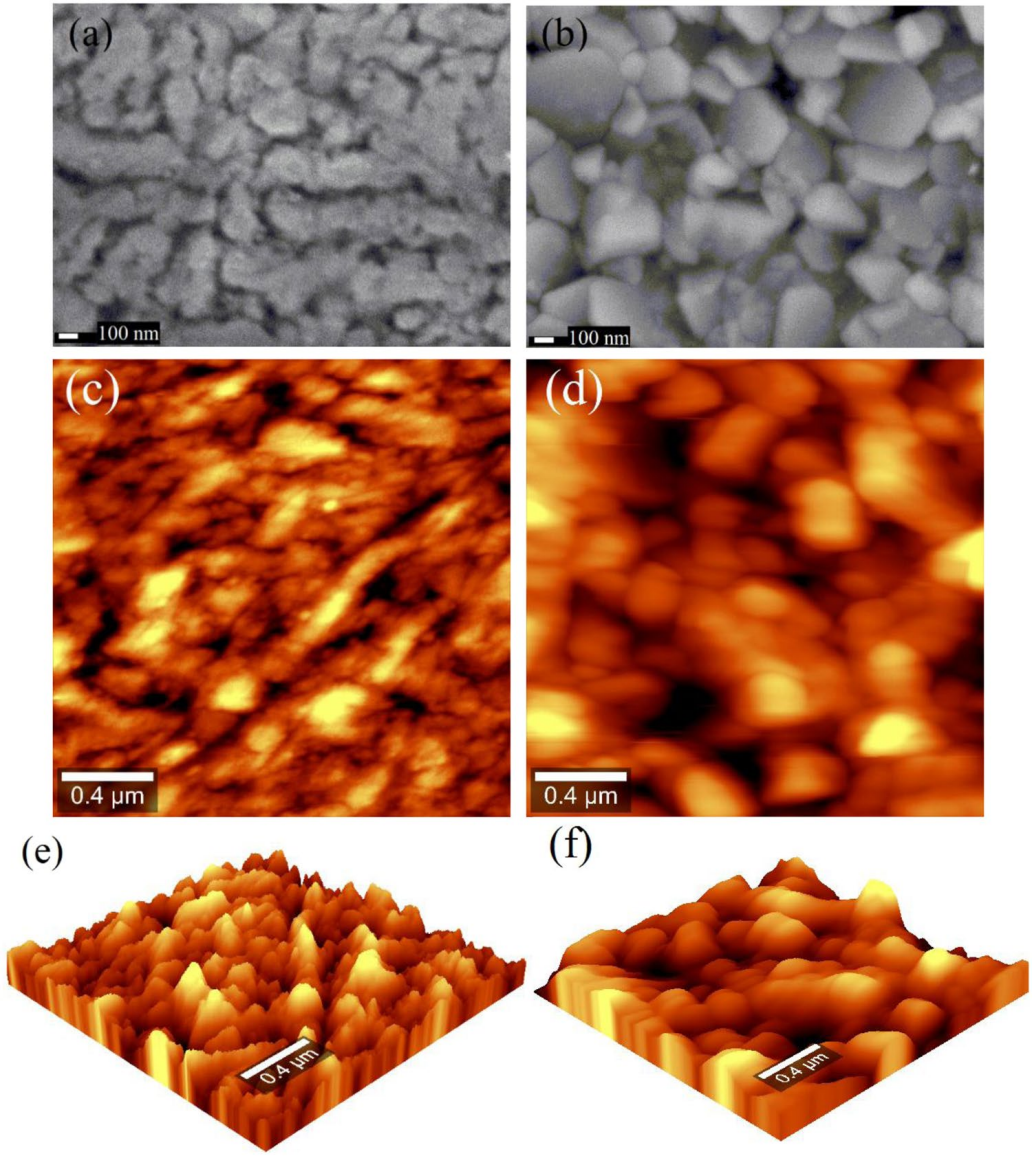
SEM micrograph of (**a**) sample 1: VO_2_ (120 nm) + r-sapphire and (**b**) sample 2: VO_2_ (300 nm) + silicon 100. Two and three-dimensional AFM images of (**c–e**) sample 1 and (**d–f**) sample 2.

The structural characterization of the samples’ surface has been performed by Field Emission Scanning Electron Microscope (FESEM JEOL 7600 F). The images obtained for the samples 1 and 2 are respectively shown in Fig. 3(a,b), which show that the grains formed on the r-sapphire substrate exhibit spherical-like and elongated shapes, such that they are well interconnected in a columnar growth. On the other hand, the grains formed on silicon present pyramidal, cubic, and sharp shapes with a connectivity relatively weaker than those on r-sapphire. These results are consistent with those obtained by an Atomic Force Microscope (AFM) integrated to a Confocal Raman Witec Alpha300 spectrometer, as shown in Fig. 3(c,d). The three-dimensional AFM plots of a 2 × 2 *μ*m^2^ are presented in Fig. 3(e,f) for both samples. The grain sizes in sample 1 varies within the range (0.12; 0.40) *μ*m, while those in sample 2 are in the interval (0.20; 0.26) *μ*m. In addition, the surface roughness of sample 1 (13.1 nm) and sample 2 (5.2 nm) have been obtained from the arithmetical mean height (5.14 nm, 14.2 nm), the root mean square height (7.2 nm, 15.7 nm) and the maximum height (28.6 nm, 106.5 nm). These morphological differences of the samples 1 and 2 are expected to have a direct impact on the effective emissivity of our VO2 films, as reported in Table 2.

**Table 2:** MIT characteristics in the emissivity of VO_2_ films grown on a sapphire (sample 1) and silicon (sample 2) substrate.

	Sample 1 (120 nm) VO_2_ + r-sapphire	Sample 2 (300 nm) VO_2_ + silicon 100	Sample 3 (300 nm) VO_2_ + r-sapphire	Sample 4 (130 nm) VO_2_ + silicon 100
Emissivity change (${\rm{\Delta }}{{\epsilon }}_{1}$)	0.45	0.42	0.34	0.27
Critical temperature ($${T}_{c})$$	64.4 °C	64.3 °C	61.2 °C	65.8 °C
Width of main hysteresis (Δ*H*)	3.3 °C	10.8 °C	2.2 °C	13.7 °C
Slope of the MIT [*β* = −${(\partial {{\epsilon }}_{1}/\partial {T}_{1})}_{{T}_{1}={T}_{c}}$]	0.08/°C (↑)	0.03/°C (↑)	0.07/°C (↑)	0.01/°C (↑)

Vertical arrows, for the slope *β*, stand for the heating process.

### Experimental setup

**Figure 4:**
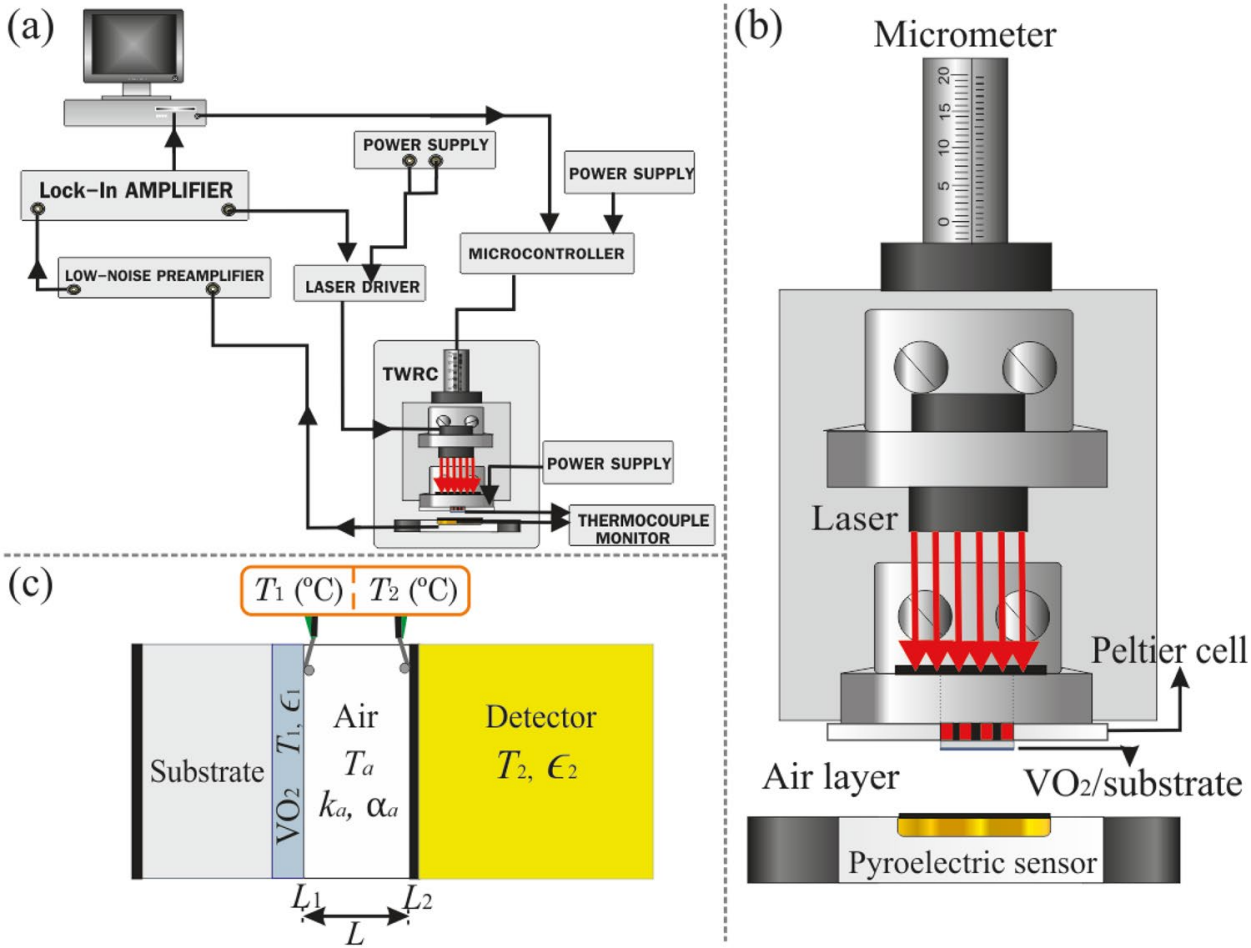
Scheme of the (**a**) experimental setup along with a (**b**) zoom-in on the resonant cavity used to monitor the photothermal signal. (**c**) Schematic illustration of the layered system undergoing heat transport.

The experimental setup used to measure the emissivity of our VO_2_ thin films consists of a thermal-wave resonant cavity (TWRC) implemented with a heating and data acquisition equipment, as shown in Fig. 4(a). The working principle of this standard photothermal technique is based on the detection of periodic temperature fluctuations (thermal waves) generated by a thin film (heater) in response to the absorption of a modulated light beam, and then transmitted through an intra-cavity fluid of thickness *L* towards a sensor placed parallel to the heater (Fig. 4(b))^51^. As the heat transport within this cavity involves both heat conduction^52,53^ and radiation^54–56^, the TWRC was applied to accurately determine the thermal diffusivity of fluids^57–60^ as well as the emissivity of solid thin films^56^ and selective solar coatings^61^. In our experimental setup, the intra-cavity fluid is air, the heat emitter is the VO_2_ + substrate sample (1.0 cm in diameter) that is heated by an infrared diode laser beam (100 mW, *λ* = 808 nm), whose spot uniformly covers all the heater and its intensity is modulated with a frequency *f* = 3 Hz. At this modulation frequency, the signal-to-noise ratio is high, even when the sample temperature increases through values within the MIT of VO_2_. The temperature of the VO_2_ + substrate sample during its heating and cooling is controlled with a Peltier cell (*V*_max_ = 5.3 V, *I*_max_ = 5.7 A, Δ*T*_max_ = 68 °C) that has a center hole allowing the incidence of the laser beam onto the sample (Fig. 2(b)). The voltage applied to the Peltier cell is switched in 0.1 V every 5 minutes, which leads to changes of about 1.7 °C in the temperature of the sample. The amplitude and phase delay of the TWRC signal are detected through a PZT pyroelectric ceramic sensor covered by a black thin layer of average absorptance 0.85 in the spectral wavelength range between 2–15 μm, positioned in front of the emitter. The pyroelectric voltage signal is fed into a preamplifier (Stanford Research Systems SR-560) and then sent into the lock-in amplifier (Stanford Research Systems SR-830 DSP) for further amplification, filtering, and demodulation. The reported signals correspond to an average of five repetitions for each fixed temperature. The cavity length *L* = 3.5 mm is set with a micrometer coupled with a stepper motor (resolution of 10 µm), while the temperatures of the sample (*T*_1_) and sensor (*T*_2_) surfaces are measured by means of K-thermocouples (0.05 mm in diameter) placed as shown in Fig. 4(c) and a thermocouple monitor (2-Channel Handheld Digital Thermometer) with 0.1 °C of resolution.

### Theoretical model

Taking into account the laser spot, modulation frequency, and cavity length used in our experimental setup, the heat propagation inside the heater (VO_2_ + substrate) and air layer, shown in Fig. 4(c), can be considered as predominantly one-dimensional. Under this condition, the normalized photothermal signal *V* recorded by the detector is given by^53^:

1 $$V(f,L,{T}_{1},\,{{\epsilon }}_{1})=\frac{{k}_{a}}{{e}_{1}{L}_{1}\sqrt{2\pi f}\,}\frac{\sqrt{{\alpha }_{1}}}{\sqrt{{\alpha }_{a}}}(\frac{1}{\sqrt{i}\,\sinh ({\sigma }_{a}L)}-i\frac{{\epsilon }}{{{\epsilon }}_{0}})$$

where *L*_1_, *e*_1_ and *α*_1_ are the thickness, thermal effusivity, and thermal diffusivity of the heater, respectively; ${\sigma }_{a}=(1+i)/{\mu }_{a}$, ${{\epsilon }}_{0}={e}_{a}\sqrt{2\pi f}/(4\sigma {T}_{1}^{3})$, ${\mu }_{a}=\sqrt{{\alpha }_{a}/\pi f}$, *α*_*a*_, *k*_*a*_ and *e*_*a*_ are the respective thermal diffusivity, thermal conductivity, and thermal effusivity of the air layer, ${\epsilon }={({{\epsilon }}_{1}^{-1}+{{\epsilon }}_{2}^{-1}-1)}^{-1}$, with ${{\epsilon }}_{1}$ and ${{\epsilon }}_{2}$ being the emissivity of the heater and the sensor, respectively, and $\sigma$ is the Stefan-Boltzmann constant. The experimental values of the amplitude and phase of *V* have been used to determine the emissivity ${{\epsilon }}_{1}$ of our VO_2_ thin films at different temperatures within their MITs.

## Results and Discussion

**Figure 5:**
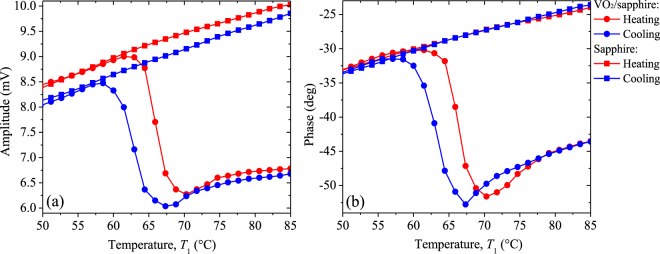
(**a**) Amplitude and (**b**) phase signals measured for the VO_2_ + sapphire sample (circles) and the sapphire substrate (squares) during the heating (red) and cooling (blue) processes.

**Figure 6:**
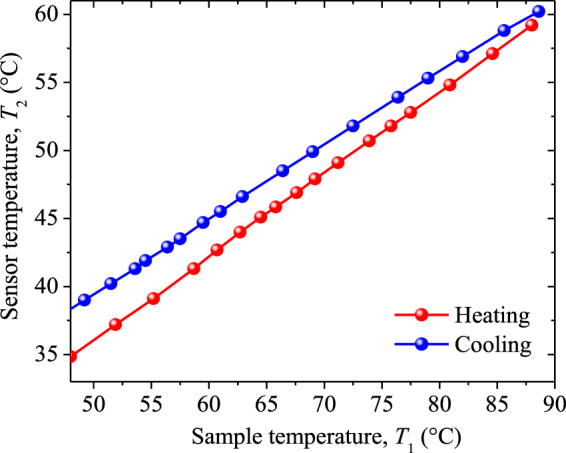
Sensor temperature as a function of the sample one, for the heating (red) and cooling (blue) processes.

**Figure 7:**
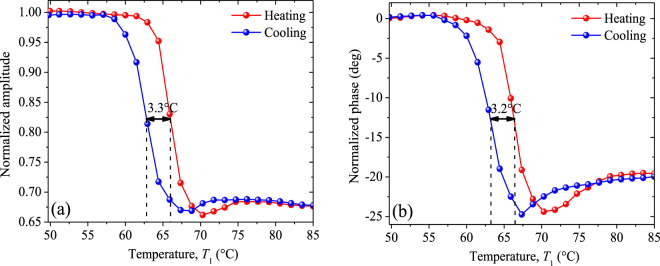
(**a**) Normalized amplitude and (**b**) normalized phase obtained during the heating and cooling of sample 1, as functions of its temperature within its MIT.

The amplitude and phase measured for the VO_2_ + sapphire and pure sapphire samples are shown in Fig. 5(a,b), respectively. Note that both signals vary linearly with the sapphire temperature during the heating and cooling processes, such that their corresponding values agree with those obtained for the VO_2_ + sapphire sample at low temperatures out of its MIT. This agreement indicates that, for these temperatures, the photothermal signals are mainly governed by the substrate and therefore no VO_2_ thin film information can be extracted from them. This confirms the fact that for such nanometric thickness, VO_2_ is fairly transparent to the infrared radiation in the dielectric phase^62^. Furthermore, during the heating process of sapphire, the amplitude takes higher values than for the cooling one, while the phase values are practically the same for both processes. The linear dependence between the temperatures of the sensor (*T*_2_) and sample (*T*_1_), with a slope for the heating process that is higher than the one for the cooling process (Fig. 6), indicates that the linear temperature dependence of the signals shown in Fig. 5 is due to the variations of *T*_2_, while the dependence (independence) of the amplitude (phase) on the heating and cooling process is due to its high (low) sensitive to the thermal energy stored by the sensor. To eliminate the dependence of the amplitude and phase on *T*_2_, and therefore on the effect of the Peltier heater, their values obtained for the VO_2_ + substrate sample are going to be normalized with the corresponding ones recorded for the substrate alone, under the same experimental conditions. The normalized signals thus obtained have the advantage of being independent of the substrate thermal properties (Eq. 1) and therefore their changes can be properly used to determine the temperature variations of the effective emissivity of the VO_2_ + substrate system, once its room temperature value is known (Table 1) and the thermal properties (*k*_*a*_, *α*_*a*_) of the intra-cavity air layer are determined from the kinetic theory of gases, for the average temperature (*T*_1_ + *T*_2_)/2. In addition, outside of the MIT, this normalization method yields photothermal signals independent of *T*_1_, as expected (Fig. 7).

**Figure 8:**
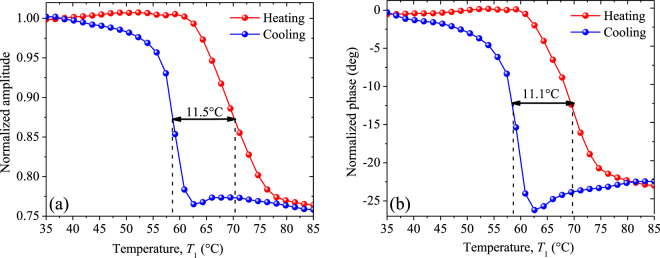
(**a**) Normalized amplitude and (**b**) normalized phase obtained during the heating and cooling of sample 2, as functions of its temperature within its MIT.

The normalized amplitude (NA) and normalized phase (NP) obtained for the sample 1 (VO_2_ + sapphire) are respectively shown in Fig. 7(a,b), as functions of its temperature *T*_1_, during the heating and cooling processes around the MIT. Within this MIT, both the NA and NP depend strongly on *T*_1_ and take different values for the heating and cooling processes, which shows that they are sensitive to the thermal hysteresis of our VO_2_ sample. The disappearance of this dependence for low and high temperatures, indicates that the sample has reached its dielectric and metallic states, respectively. The significant differences of the NA and NP between these states are of 34% and 24.4 degrees, respectively, and involve a characteristic hysteresis cycle on both signals. A similar behavior is exhibited by the NA and NP shown in Fig. 8(a,b) for the sample 2 (VO_2_ + silicon), however, the hysteresis width (11.5 °C) at mid height of the normalized amplitude, for this latter sample, is significantly wider than that (3.3 °C) of the former one. These sizeable differences on the hysteresis width also show up on the normalized phase and are reasonable due to the fact that the VO_2_ film growth orientation along with the shape and distribution of the film grains, are mainly driven by the substrate material, as shown in Figs 2 and 3 as well as in the supplementary material. The silicon substrate of VO_2_ films thus enables an epitaxial growth and microstructure that generate a wider thermal hysteresis than sapphire.

**Figure 9:**
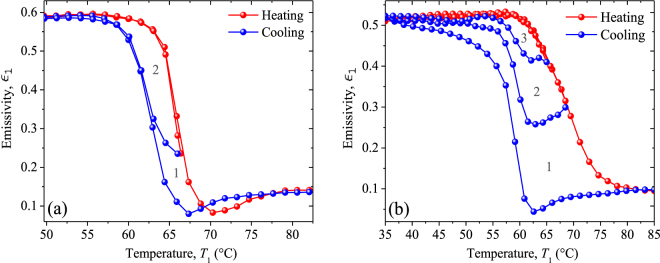
Principal (1) and secondary (2, 3) hysteresis loops of the emissivity of VO_2_ thin films grown on (**a**) r-sapphire and (**b**) silicon (100) substrates.

Figure 9(a,b) show the emissivity ${{\epsilon }}_{1}$ of our VO_2_ thin films deposited on a substrate of sapphire (sample 1) and silicon (sample 2), respectively. In the MIT, ${{\epsilon }}_{1}$ decreases during the heating process and increases for the cooling one, following a different path, which leads to a hysteresis loop similar to that of its corresponding photothermal signals shown in Figs 7 and 8. The significant variations of ${{\epsilon }}_{1}$ from 0.59 (0.52) in the insulating phase, to 0.13 (0.10) in the metallic one, of sample 1 (2), are consistent with previous results reported in the literature for VO_2_ thin films grown on a sapphire^30,31,44^ (silicon)^45–48^ substrate. However, in contrast to these seven-latter works based on conventional reflectance/transmittance spectroscopy operating in a limited spectral range (2–15 μm), our direct measurements of the VO_2_ emissivity ${{\epsilon }}_{1}$ are expected to be more accurate, as the thermal-wave resonant cavity is able to detect radiation in a much broader spectra range. The effect of the MIT on the emissivity of each sample can be characterized by the emissivity change ${\rm{\Delta }}{{\epsilon }}_{1}$, critical temperature of transition (*T*_c_), width (Δ*H*), and slope ($\beta$); which are summarized in Table 2. Note that samples 1 and 2 have similar values for ${\rm{\Delta }}{{\epsilon }}_{1}$ and ${T}_{c}$, but sizeable differences on ${\rm{\Delta }}H$ and $\beta$. Sample 2 exhibits a parameter ${\rm{\Delta }}H$ ($\beta$) around 3 times greater (smaller) than that of sample 1. These differences can be attributed to the growth orientation and the surface morphology of our VO_2_ thin films, which depend on their respective substrate^39,48–50^ and film thickness^47,50^, as shown in Figs 2 and 3. Taking into account that the thin films deposited by the PLD technique can be considered as a collection of grains (Fig. 3), which in the VO_2_/sapphire sample exhibit a stronger coupling that in the VO_2_/silicon, and that a crystal requires less energy to change its phase when their grains are more coupled, the Δ*H* (*β*) of this latter sample should be wider (slower) than that of the former one, as reported in Table 2. Note that Table 2 also shows that the width Δ*H* and slope *β* of the emissivity of samples with the same substrate (samples 1 and 3 or samples 2 and 4) do not change significantly with the sample thickness, which confirms that the values of these parameters are mainly determined by the substrate. This fact is further supported by Figs 3 and S7, which show that the film substrate has a stronger impact than the film thickness, on the shape and distribution of grains. It is therefore clear that a silicon substrate yields wider hysteresis loops with slower phase transition rates than sapphire. This result may be used, for instance, in optical memory-type applications, which require a large Δ*H*^63^, and for the development of VO_2_-based bolometers, in which a small Δ*H* is preferable^64^.

Secondary hysteresis loops (2, 3), shown in Fig. 9(a,b), have been obtained by interrupting the heating process of both samples at a temperature *T*_*f*_ within their MITs. These mini loops span inside the principal ones (1), especially for sample 2, which has a wider hysteresis loop (1) than sample 1. Note that the cooling branches of the three loops recorded for sample 2 follow different paths, such that they reach the insulating-state emissivity at different temperatures *T*_0*i*_ (Fig. 9(b)). Lower *T*_*f*_ generates a hysteresis loop with higher *T*_0*i*_ and therefore smaller Δ*H*. Given that the slope *β* of the emissivity also decreases as *T*_*f*_ (or Δ*H*) reduces, during the cooling process, the hysteresis width, transition rate, and variation of the VO_2_ emissivity can be cut off by interrupting the heating process at a low enough *T*_*f*_. On the other hand, note that the minimum of the principal hysteresis loop of sample 2 also shows up on their secondary ones, but tends to disappear as VO_2_ moves away from its metallic state (lower *T*_*f*_). These minima are consistent with previous observations^46^ and can be associated to the anomalous energy absorption of VO_2_.

### Application in thermal rectification

**Figure 10:**
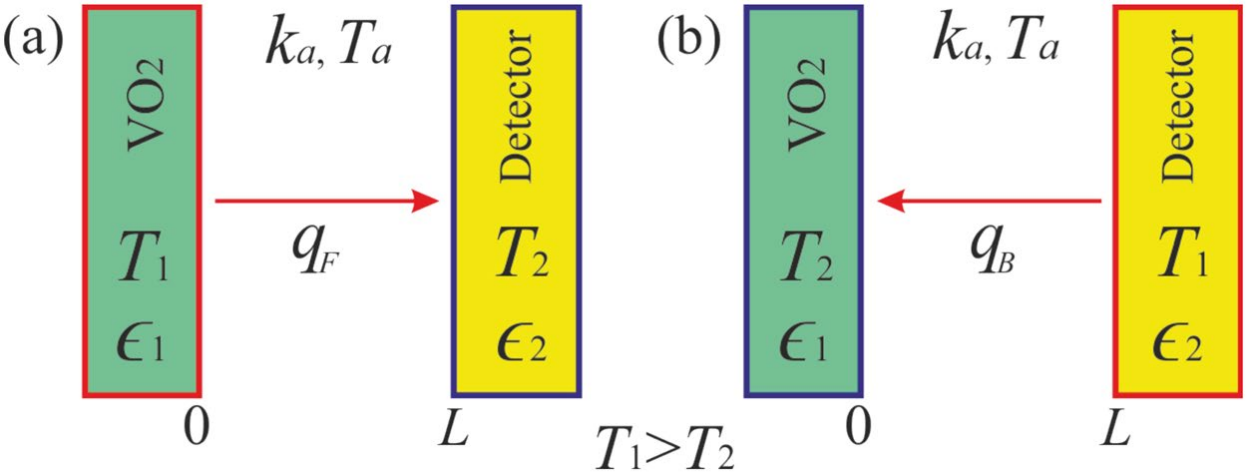
Scheme of a thermal diode operating in the (**a**) forward and (**b**) backward configurations.

The experimental variations of the VO_2_ emissivity ${{\epsilon }}_{1}$(*T*) with temperature are now applied to assess the rectification factor of the radiative thermal diode operating in the forward and backward configurations shown in Fig. 10(a,b). This diode essentially consists of a TWRC (Fig. 4(c)), whose terminals are set at temperatures ${T}_{1}$ and ${T}_{2}\,(\, < \,{T}_{1})$ and are separated by an air layer of thermal conductivity ${k}_{a}({T}_{a})$, with ${T}_{a}=({T}_{1}+{T}_{2})/2$. Taking into account the conductive heat flux ${q}_{C}={k}_{a}({T}_{1}-{T}_{2})/L$ propagating through the air between the diode terminals, the net heat flux in the forward (${q}_{F}$) and backward (${q}_{B}$) bias are given by

2a $${q}_{F}=\frac{{k}_{a}}{L}({T}_{1}-{T}_{2})+{\epsilon }({T}_{1})\sigma ({T}_{1}^{4}-{T}_{2}^{4}),$$

2b $${q}_{B}=\frac{{k}_{a}}{L}({T}_{1}-{T}_{2})+{\epsilon }({T}_{2})\sigma ({T}_{1}^{4}-{T}_{2}^{4}),$$

where ${\epsilon }(T)={({{\epsilon }}_{1}^{-1}(T)+{{\epsilon }}_{2}^{-1}-1)}^{-1}$. For the sake of simplicity, we are going to consider that the detector has a temperature independent emissivity ${{\epsilon }}_{2}=0.85$, which is the case of the detector used to perform our experiments (Fig. 4(c)).

According to Fig. 9(a,b), the emissivity ${{\epsilon }}_{1}({T}_{1}) < {{\epsilon }}_{1}({T}_{2})$ for most temperatures ${T}_{1} > {T}_{2}$, which indicates ${q}_{B} > {q}_{F}$. The rectification factor $R=1-{q}_{F}/{q}_{B}$ can then be written as follows:

3a $$R=\frac{1}{1+N}(1-\frac{{{\epsilon }}_{1}({T}_{1})}{{{\epsilon }}_{1}({T}_{2})}),$$

3b $$N=\frac{{k}_{a}}{\sigma L}\frac{1}{{{\epsilon }}_{1}({T}_{2})({T}_{1}+{T}_{2})({T}_{1}^{2}+{T}_{2}^{2})}$$

Equations (3a) and (3b) establish that, in absence of heat conduction (${k}_{a}=0$), $N=0$ and *R* = *R*_*max*_ = $1-{{\epsilon }}_{1}({T}_{m})/{{\epsilon }}_{1}({T}_{i})$ takes its maximum value, when the terminal temperatures ${T}_{1}$ and ${T}_{2}$ are chosen as the minimal (${T}_{m}$) and maximal (${T}_{i}$) ones supporting the metallic and insulator phases, respectively. By contrast, in presence of heat conduction (${k}_{a} > 0$), the maximum rectification is reached for ${T}_{2}={T}_{i}$ and ${T}_{1}\gg {T}_{m}$.

**Figure 11:**
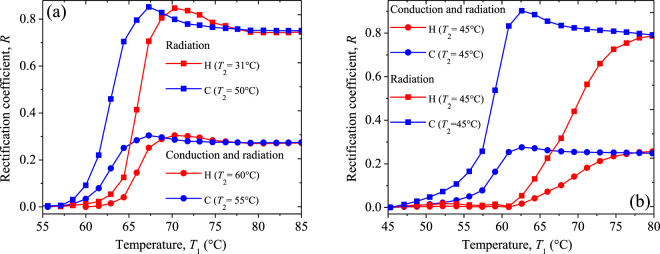
Rectification factor *R* as a function of the temperature *T*_1_ of a thermal diode made up with a terminal of VO_2_ present in (**a**) sample 1 and (**b**) sample 2. Calculations have been done for pure radiation (squares) and radiation + conduction (circles) heat transfer, by choosing the temperature *T*_2_ that maximizes *R* during the heating and cooling processes. The letters *H* and *C* stands for the heating and cooling processes, respectively.

Figure 11(a,b) show the rectification factor *R* as a function of the temperature *T*_1_ of the thermal diode (Fig. 10) operating with a VO_2_ terminal made up of sample 1 or sample 2, respectively. The general increase of *R* with *T*_1_, within the MIT of both samples, is the result of the decrease of ${{\epsilon }}_{1}$ when their temperatures rise, as shown in Fig. 9(a,b). In all cases, higher rectifications are obtained when the temperature *T*_1_ (*T*_2_) of the hotter (cooler) diode terminal is chosen in the metallic (insulating) phase of VO_2_, as expected. In this case, the asymptotic value of the rectification factor *R* of a thermal diode in vacuum (no heat conduction) is 0.73, for sample 1 and 0.78 for sample 2. By contrast, in presence of heat conduction, these values reduce to about 0.26 for both samples. This reduction is reasonable, given that the asymmetry between the heat fluxes ${q}_{F}$ and ${q}_{B}$ is only driven by the temperature dependence of the VO_2_ emissivity and not by the heat conduction through air, as established by Eqs (3a) and (3b). In presence of pure heat radiation, the highest rectification factor *R* = 0.87 occurs at the temperature *T*_1_ for which the VO_2_ emissivity exhibits a minimum and is reached for a temperature difference Δ*T* = *T*_1_ − *T*_2_ = 17.3 °C, in both samples. This record-breaking rectification factor, for a relatively small temperature change of the diode terminals, shows the high suitability of VO_2_ for developing efficient radiative thermal diodes.

## Conclusions

We have experimentally measured the principal and secondary hysteresis loops of the emissivity of two VO_2_ thin films grown on r-sapphire and silicon (100) substrates. These first-time-ever direct measurements have been performed through the high-accurate technique of the thermal-wave resonant cavity, which involves both thermal radiation and heat conduction. Remarkable VO_2_ emissivity variations up to 43% have been observed at the extremes of the metal-insulator transition of both VO_2_ films. Based on a PLD process, it has been shown that the silicon substrate induces the growth of VO_2_ films with three-time slower phase transitions and wider principal hysteresis loops than a sapphire one. We have also found that the hysteresis width and the transition rate of the VO_2_ emissivity can be tuned by means of the secondary hysteresis loops. Furthermore, the potential of VO_2_ for building up a radiative thermal diode with a record-breaking rectification factor up to 87%, with a relatively small temperature difference of 17.3 °C between its terminals, has been demonstrated.

## Electronic supplementary material

Supplementary information
