# Supplementary material for: Thermal hysteresis measurement of the VO2 emissivity and its application in thermal rectification
Source: Sci Rep. 2018 May 31;8:8479. doi: 10.1038/s41598-018-26687-9 (PMC5981426; doi:10.1038/s41598-018-26687-9)
Supplement: Supplementary file 1 — Supplementary information [file 41598_2018_26687_MOESM1_ESM.pdf]

Thermal hysteresis measurement of the VO_2 emissivity and its application in thermal rectification

C.L. Gomez-Heredia[1,2], J. A. Ramirez-Rincon[1,2], J. Ordonez-Miranda[2*], O. Ares[1], J. J. Alvarado-Gil[1,2], C. Champeaux[3], F. Dumas-Bouchiat[3], Y. Ezzahri[2], K. Joulain[2]

[1] Departamento de Física Aplicada, Cinvestav-Unidad Mérida, Carretera Antigua a Progreso km. 6, 97310, Mérida, Yucatán, México.

[2] Institut Pprime, CNRS, Université de Poitiers, ISAE-ENSMA, F-86962 Futuroscope Chasseneuil, France.

[3] Université de Limoges, CNRS, IRCER, UMR 7315, F-87000 Limoges, France.

*Corresponding author: jose.ordonez@cnrs.pprime.fr

Average emissivity calculation at room temperature

According to the principle of energy conservation and the Kirchhoff law, the spectral emissivity of materials with reflectance $r(\lambda)$ and transmittance $t(\lambda)$ is given by: $1 - r(\lambda) - t(\lambda)$. The average emissivity ϵ_1 over the spectral wavelengths λ of our samples at room temperature ($T = 300$ K) can then be determined by

$$\epsilon_1 = \frac{\int_0^\infty [1-r(\lambda)-t(\lambda)]\Theta(\lambda,T)\,d\lambda}{\int_0^\infty \Theta(\lambda,T)\,d\lambda} = \frac{1}{\sigma T^4}\int_0^\infty [1-r(\lambda)-t(\lambda)]\Theta(\lambda,T)\,d\lambda, \qquad \text{(S1)}$$

where $\Theta(\lambda,T) = C_1\lambda^{-5}/(e^{C_2/\lambda T}-1)$ is the blackbody spectral radiance, $C_1 = 1.48\times 10^{-15}$ Wm^2, $C_2 = 1.43\times10^{-2}$ m $\cdot$ K, and $\sigma = 5.67\times10^{-8}$ Wm^{-2}K^{-4}. To determine the numerical value of ϵ_1, the reflection and transmission spectra of samples 1 and 2 have been measured for wavelengths between 2 μm and 22 μm, by means of a PerkinElmer Frontier FTIR spectrometer. The results are shown in Figure S1.

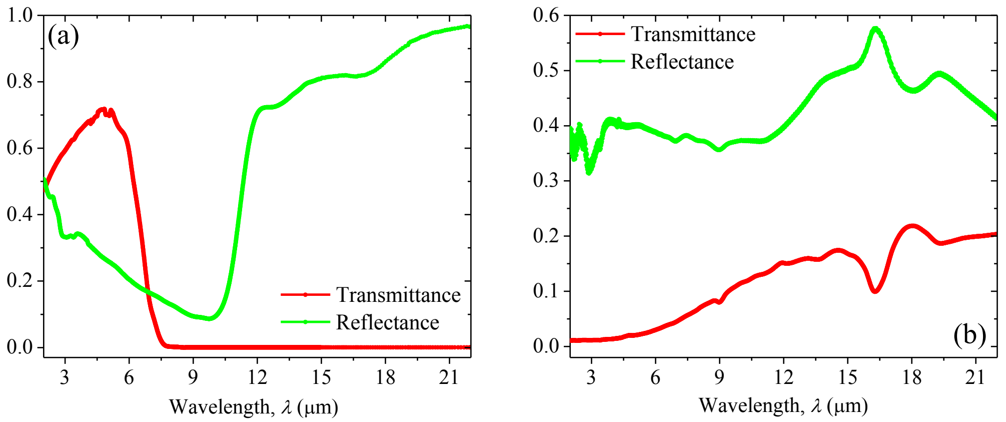

Figure S1. Experimental transmittance and reflectance spectra of (a) sample 1 and (b) sample 2 at room temperature, as functions of the spectral wavelength.

According to the ratio of emitted power $REP = [1 - r(\lambda) - t(\lambda)]\Theta(\lambda, T)/\sigma T^4$ shown in Fig. S2, the main contributions to the integrant in Eq. (S1), and therefore to the average emissivity ϵ_1, come from wavelengths between 5 μm and 15 μm. This indicates that the spectral range of 2-22 μm is wide enough of calculate the integral in Eq. (1) and thus to determine ϵ_1 with a fairly good accuracy, specially for sample 1. The final results are, $\epsilon_1 = 0.59$ for sample 1 and $\epsilon_1 = 0.52$ for sample 2.

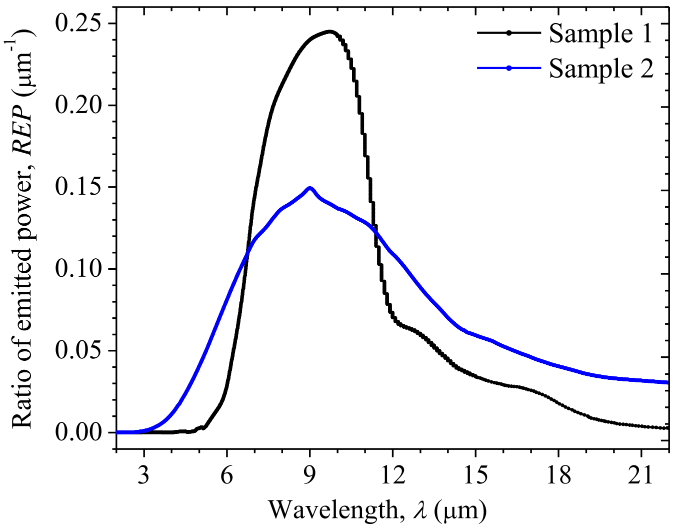

Figure S2. Ratio of emitted power (*REP*) of sample 1 (black line) and sample 2 (blue line) at room temperature.

Effects of the deposition process, film thickness, and substrate on the VO_2 films

In a bottom-up approach, the characteristics of thin films with thicknesses up to 1 μm, are strongly influenced by the deposition process as well as by the nature and temperature of their substrates. For a crystallized substrate, the classical three primary deposition modes: *Volmer–Weber* (3D ad-atom clusters = islands), *Frank–van der Merwe* (2D ad-atom = layers) and *Stranski-Krastanov* (layer + island growth, a mixture of the first two) can be applied to epitaxially grows thin films at a crystal surface or interface. For the case of the PLD process used in this work, the material deposition is done out of thermodynamic equilibrium and in a strong directional way, which favours the appearance of islands (grains) and columnar growth.

The two substrates r-sapphire and silicon used in this work are different regarding the nature elements and the atomic structures. r-sapphire is a single crystal of Al_2O_3 cut in a specific orientation $(1\bar{1}02)$, with a very smooth surface (roughness peaks less than 0.3 nm). c- and r-sapphire are ideal substrates for VO_2 growth due to the relatively small lattice mismatch. O the other hand, the Si(100) substrate constitutes a single crystal cut through the crystal exposing the (100) surface. An amorphous native oxide layer with a thickness of about ~5 nm covers the Si(100) surface.

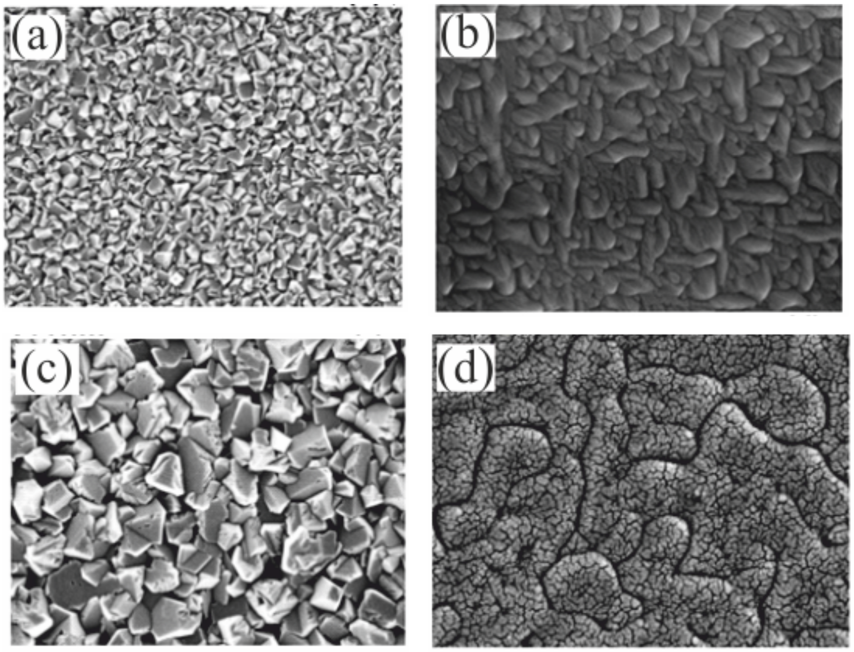

Figure S3. SEM (1.5µm × 2.0µm) images obtained for four VO_2 thin films (top views) synthesized using identical PLD parameters. (a) and (c) stand for two VO_2 films deposited on a native-SiO_2/Si substrate and show grains with sharp shapes. (b) and (d) represent two VO_2 films deposited on an r-sapphire substrate and display flat grains with an in-plane growth. The cracks observed in (d) are due to a defect of the platinum deposition during the metallization process used for obtaining the SEM images. The size of the grains is thus a function of VO_2 film thickness but their shape is determined by the substrate mainly.

According to Figs. S3 and S4, the substrates of r-sapphire and SiO_2/Si lead to the formation of clusters of atoms (grains) with different shapes and distributions. The separated grains developed on SiO_2/Si exhibit pyramidal, cubic, elongated, and sharp shapes with a disorganised distribution due essentially to the amorphous SiO_2 nanolayer. By contrast, the grains formed on r-sapphire present elongated and spherical-like shapes, and their sizes in the (*xy*) substrate plan are larger than the ones growth on SiO_2/Si. Furthermore, the degree of connectivity of the grains on r-sapphire is higher than the one of the grains on SiO_2/Si, as confirmed by the AFM images shown in Fig.3. The r-sapphire substrate tends to organize the grains and lead to low roughness (Sa=4.98 nm, Sq=6.18 nm, Sz=42.30 nm, ISO25178), while the SiO_2/Si allows the growth of grains in the x, y and z directions, which leads to a relatively high roughness (Sa=12.30 nm, Sq=15.40 nm, Sz=105.00 nm, ISO25178) with a peak-to-peak distance (105 nm) comparable to the film thickness (100 nm). The SiO_2 amorphous layer is thus the source of the individual grains with random distribution shown in Figs. S3 and S4.

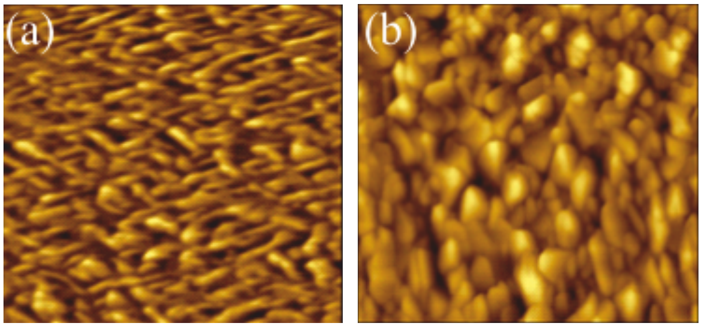

Figure S4. AFM (2.0 µm × 2.0 µm) images obtained for two VO_2 thin films (100 nm) growth on substrates of (a) r-sapphire and (b) SiO_2/Si. The average peak-to-peak distance (105.0 nm,) in (a) is longer than that (42.3 nm) in (b), which indicates that the roughness of the sample in (a) is higher than the one of the sample in (b).

Microstructures of the VO_2+r-sapphire samples determined by SEM and AFM are consistent with the structure (atomic arrangement) provided by the XRD patterns shown in Fig. S5, where the VO_2 peaks are clearly visible. For the two thinner samples (100 nm and 200 nm in thicknesses), the VO_2 layers exhibit a mono-orientation characterized by (200) and (400) diffraction peaks, which is compatible with the grain shapes shown by both the SEM and AFM images in Figs. S3 and S4. An addition orientation (111) appears for the 400 nm-thick sample, which shows that as the film thickness increases, the substrate influence reduces and the material structure tends to depend more on the VO_2 intrinsic properties, as expected.

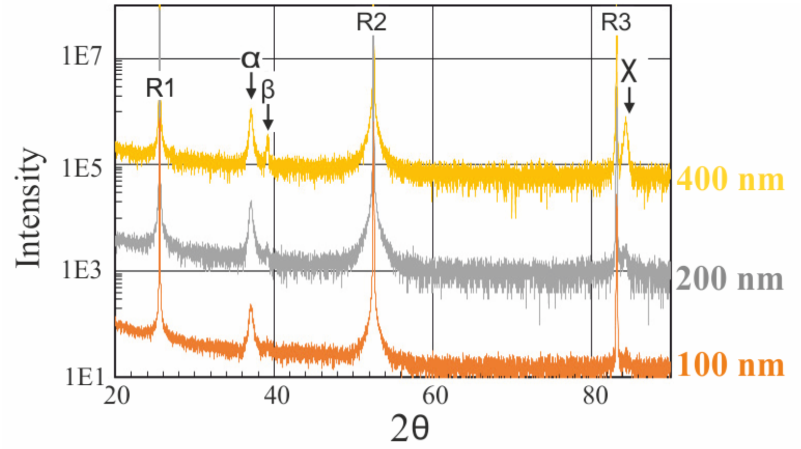

Figure S5. XRD (θ, 2θ) patterns obtained for three VO_2 thin films synthesized on an r-sapphire substrate with thicknesses of 100 nm, 200 nm, and 400 nm. The diffraction peaks (α, β, X) = ((200), (111), (400)) and (R_1, R_2, R_3) = ((012), (024), (036)) are associated to VO_2 and substrate, respectively.

Figure S6 show that the XRD patterns obtained for VO_2 deposited on SiO_2/Si are different than those shown in Fig. S5 for VO_2 deposited on r-sapphire. For the 100 nm-thick sample, there are not clear diffraction peaks, which indicates that the diffracting objects (grains) are

too small and that their atomic order is weak. By contrast, the 300 nm-thick VO_2 film does present the diffraction peaks (011) and (022), which proves the mono-orientation in the sample.

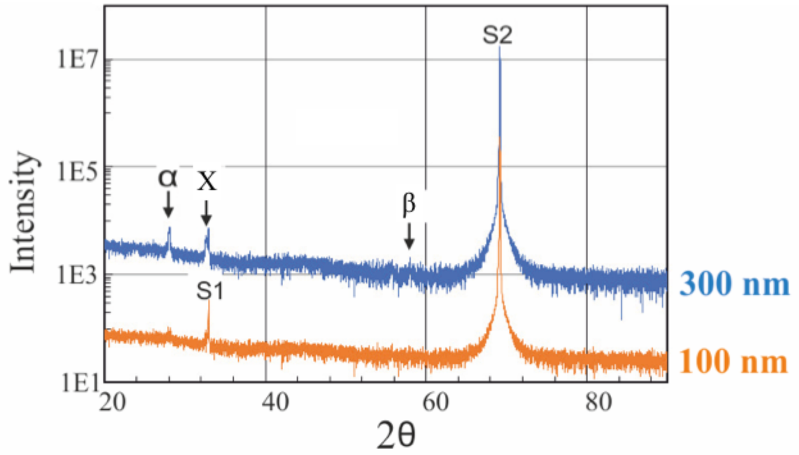

Figure S6. XRD (θ, 2θ) patterns obtained for two VO_2 thin films synthesized on a native-SiO_2/Si substrate with thicknesses of 100 nm and 300 nm. The diffraction peaks (α, β) = ((011), (022)), X = (301), and (S_1, S_2) = ((002), (004)) are associated to VO_2, V_2O_5, and substrate, respectively.

SEM, AFM, and XRD images show thus significant differences between VO_2 films deposited on r-sapphire and SiO_2/Si. The substrate generally drives the grained microstructure of VO_2 films with thickness smaller than 500 nm, while thicker films are expected to exhibit a reinforcement of the intrinsic VO_2 materials properties.

Characterization of the complementary samples 3 and 4

In order to properly compare the shape and distribution of grains of our samples 1 (VO_2 (120 nm)+r-sapphire) and 2 (VO_2 (300 nm)+silicon (100)) with those of VO_2 films with different substrates and comparable thicknesses, the SEM images of our complementary samples 3 (VO_2(300 nm)+r-sapphire) and 4 (VO_2 (130 nm)+silicon (100)) are shown in Fig. S7.

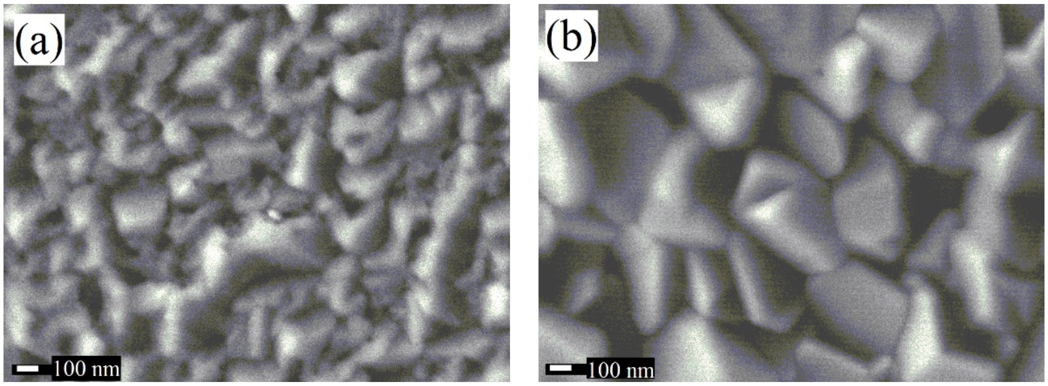

Figure S7. SEM micrographs of the samples (a) 3: VO_2(300 nm)+r-sapphire and (b) 4: VO_2 (130 nm)+silicon (100).

According to Figs. 3 and S7, the degree of connectivity of the grains in the samples 1 and 3 deposited on an r-sapphire substrate is higher than the corresponding ones of the grains in the samples 2 and 4 deposited in silicon (100). This structural difference along with the disparities on the grains' geometry hold for two different films with the same substrate and different thicknesses (in the range between 120 and 300 nm) indicate that the substrate has a stronger effect than the film thickness, on the MIT features of VO_2. This is the reason why the hysteresis width ΔH and slope β of the VO_2 emissivity are different for samples with different substrates, as reported in Table 2, within the article.

Sensor temperature dependences

By plotting the sensor temperature as a function of the sample temperature (See Fig. S8), we have found a linear dependence between both temperatures. The slope of the lines depends on the cavity length L as well as on heating and cooling procedures, but it is completely independent of the modulation frequency, which shows that this is a net effect of the changes in the stationary component of temperature. For various values of L, we found that for the cooling process this slope is lower than that for the heating one. This indicates that the sensor (PZT ceramic) used in our experiments has a high thermal inertia and store a high amount of thermal energy during the heating process that is slowly released during the cooling one. This is the reason why, the amplitude, which is directly related to the amount of thermal energy transported through the cavity, during the heating process is higher than during the cooling one. On the other hand, the phase signal represents the delay between the generated and detected thermal waves and is not affected by the thermal energy stored by the sensor, as shown in Fig. 5(b).

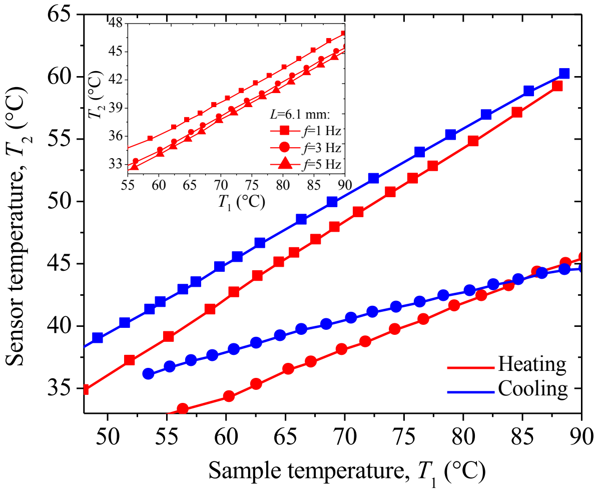

Figure S8. Sensor temperature versus sample temperature obtained for the heating (red) and cooling (blue) processes of sample 1. Experiments have been performed at f=3 Hz and two cavity thicknesses of L=3.5 mm (squares) and L=6.1 mm (circles). In the inset T_1 vs T_2 for a fixed L=6.1 mm and three frequencies of f=1 Hz (squares), f=3 Hz (circles) and f=5 Hz (triangles).
